# Method for Extracting Optical Element Information Using Optical Coherence Tomography

**DOI:** 10.3390/s24216953

**Published:** 2024-10-30

**Authors:** Jiucheng Nie, Yukun Wang, Dacheng Wang, Yue Ding, Chengchen Zhou, Jincheng Wang, Shuangshuang Zhang, Junwei Song, Mengxue Cai, Junlin Wang, Zhongxu Cui, Yuhan Hou, Si Chen, Linbo Liu, Xiaokun Wang

**Affiliations:** 1 Changchun Institute of Optics, Fine Mechanics and Physics, Chinese Academy of Sciences, Changchun 130033, China; niejiucheng22@mails.ucas.ac.cn (J.N.); wangdacheng21@mails.ucas.ac.cn (D.W.); 13384494452@163.com (Y.D.); zhouchengchen23@mails.ucas.ac.cn (C.Z.); wangjincheng@ciomp.ac.cn (J.W.); zhangshuangshuang@ciomp.ac.cn (S.Z.); sjw971222@163.com (J.S.); caimengxue1998@163.com (M.C.); wangjl@ciomp.ac.cn (J.W.); cuicuicui388@sohu.com (Z.C.); houyuhan@ciomp.ac.cn (Y.H.); 2 University of Chinese Academy of Sciences, Beijing 100039, China; 3 State Key Laboratory of Applied Optics, Changchun 130033, China; 4 Key Laboratory of Advanced Manufacturing for Optical Systems, Chinese Academy of Sciences, Changchun 130033, China; 5 School of Electrical and Electronic Engineering, Nanyang Technological University, Singapore 639798, Singapore; chensi@ntu.edu.sg (S.C.); liulinbo@ntu.edu.sg (L.L.)

**Keywords:** spectral-domain OCT, thickness measurement, curvature measurement, multilayer film

## Abstract

This study examines the measurement of film thickness, curvature, and defects on the surface or inside of an optical element using a highly accurate and efficient method. This is essential to ensure their quality and performance. Existing methods are unable to simultaneously extract the three types of information: thickness, curvature, and defects. Spectral-domain optical coherence tomography (SD-OCT), a non-invasive imaging technique with imaging depths down to the millimeter scale, provides the possibility of detecting the optical element components’ parameters. In this paper, we propose an error correction model for compensating delay differences in A-scan, field curvature, and aberration to improve the accuracy of system fitting measurements using SD-OCT. During data processing, we use the histogram-equalized gray stretching (IAH-GS) method to deal with strong reflections in the thin film layers inside the optics using individual A-scan averages. In addition, we propose a window threshold cutoff algorithm to accurately identify defects and boundaries in OCT images. Finally, the system is capable of rapidly detecting the thickness and curvature of film layers in optical elements with a maximum measurement depth of 4.508 mm, a diameter of 15 × 15 mm, a resolution of 5.69 microns, and a sampling rate of 70 kHz. Measurements were performed on different standard optical elements to verify the accuracy and reliability of the proposed method. To the best of our knowledge, this is the first time that thickness, curvature, and defects of an optical film have been measured simultaneously, with a thickness measurement accuracy of 1.924 µm, and with a difference between the calibrated and nominal curvature measurements consistently within 1%. We believe that this research will greatly advance the use of OCT technology in the testing of optical thin films, thereby improving productivity and product quality.

## 1. Introduction

The performance and stability of an optical element depend heavily on the accurate control of its surface and internal structure. It is important to detect film thickness, curvature, and defects on the surface or inside of optical elements. Firstly, the accurate measurement of the thickness ensures optimal optical characteristics of the optics, thus improving the overall performance of the system. Second, the precise control and detection of the curvature help to maintain the focusing ability and imaging quality of the optics. Finally, detecting and repairing defects (such as microcracks, bubbles, and impurities) extends the life of the optics. These inspection processes are essential for manufacturing high-quality optical components and ensuring reliability in their applications. At present, methods for detecting thickness, curvature, and defects in optical components are classified as contact or non-contact. Two primary technical approaches involving contact were pioneered by Professor Calvin Quate’s team at Stanford University in collaboration with IBM, resulting in the invention of the atomic force microscope (AFM) in 1986 [1]. Additionally, the first commercially accessible scanning electron microscope (SEM) was introduced by Cambridge Instruments in the UK in 1965 [2]. These methods discussed are proficient in acquiring images that provide insights into the topography and composition of the sample. However, it is easy to damage the surface during inspection and requires significant time costs. Moreover, it is not possible to perform real-time in situ inspection during the nominal machining process. Several non-contact methods currently in use will be described below. The two-beam laser interferometry method is a technique that utilizes a single-frequency laser for detection [3]. For example, the CHRocodile 2 IT interferometer from Prezt, Germany, enables fast and precise measurements of transmissive industrial materials. The instrument collects scattered photons from the upper and lower surfaces of the sample and derives the thickness from the interference intensity. The method has the advantages of high measurement accuracy and efficiency, and the combination of a 2D linear stage provides accurate curvature information. Its scanning speed and field of view are small. However, this method can improve the field of view if a telecentric scanning objective with a large field of view is used, which is a future research direction. Ellipsometry obtains thickness and surface shape information by detecting changes in the polarization state of the reflected light from the sample [4]. The elliptical polarization spectrometer developed by Horiba Scientific in France uses this principle. Linearly polarized light changes its state of polarization after reflection from a thin film sample. Since the polarization state of the reflected light is highly sensitive to thickness variations, the method can achieve nanoscale measurement accuracy. However, their measurement range is usually limited to a few micrometers. In addition, a series of parameters need to be adjusted for each measurement, which makes this method relatively slow [5]. X-ray resolution can reach the nanometer level and plays an important role in chip failure analysis [6]. However, X-rays cannot acquire depth information at the millimeter depth scale and cannot measure fitted curvature information. Computed tomography (CT) and ultrasound imaging can obtain cross-sectional imaging of the film, but axial resolution is limited to detecting defects in the sample layer [7,8].

SD-OCT technology is the second generation of optical coherence tomography, which employs high-speed linear arrays of charge-coupled devices (CCDs) at frequencies up to hundreds of thousands of hertz. Its single-point measurement is highly efficient and can be combined with other techniques such as adaptive optics [9]. The curvature, thickness, and defects of the sample are measured simultaneously and accurately at high speeds [10,11,12]. This technique employs broadband light to detect the sample and analyze the interference signal produced by backscattered light from the sample and reference light to reveal the internal structure of the sample. Consequently, interferometry can enhance weak signals with a superior signal-to-noise ratio in contrast to intensity detection techniques. OCT methods are widely utilized in various medical disciplines including ophthalmology [13,14,15], otology [16], dermatology [17], and brain research [18]. The distinct imaging characteristics of OCT systems meet inspection requirements in diverse fields, leading to their application in industrial sectors like LCDs, printed electronics, and composites [19,20,21]. SD-OCT can fulfill most of the requirements for thickness and curvature detection [22,23,24,25]. Dunkers applied OCT to highly scattering materials (e.g., polymer composites) and evaluated OCT as an imaging technique for fiber bundles and voids in different materials. The quality of the OCT images was found to be severely affected by the refractive index mismatch between the fibers and the reinforcement material. However, the article does not give a detailed solution for image processing, and the quality of the final images obtained remains very unclear [26]. Frank et al. applied full-field-of-view optical coherence tomography (FF-OCT) in the evaluation of optical substrate SSDs. The difficulty in observing and evaluating SSDs is that the low signal strength of SSDs disappears under noise. However, there is not much quantitative analysis in this paper; the exact amount of actual thickness is not given and no error analysis is conducted [27]. Wolfgang et al. used optical coherence tomography to analyze non-invasive monitoring of membrane thickness in a coextruded drug delivery system. Membrane properties of ethylene vinyl acetate (EVA)-based coextruded materials were evaluated, but the samples used were not multilayer membrane structures with more than two membranes [28]. Measurement and correction of sectorial aberration in optical coherence tomography (OCT) is discussed by Ortiz et al. [29]. Its relation to scanner mirror separation and collimating lens design is evaluated, and the optimal axial position of this lens is estimated to minimize sectorial aberration. However, the paper only presents the correction model without showing the final image or comparing the error of the image before and after correction from an image perspective. Manalla employed the OCT technique to measure the thickness of monolayer films, extracting film thickness from OCT signals in Fourier-transformed k-space. However, they have studied 3D imaging of multilayer films [30]. MF Shirazi conducted an industrial inspection of defects in multilayer optical films on touchscreen panels using line-field SD-OCT. Nonetheless, there is no analysis provided regarding film-related thickness and surface shape [31]. 

This paper presents an error correction model. It is used to detect the film thickness of optical elements, as well as the curvature and defects on the surface or inside with high accuracy and efficiency. The error correction model is used to compensate for the A-scan time delay, field curvature, and other aberrations, thus improving the accuracy of the system fitting measurements. The extraction method considers the actual light source dispersion and uses the group refractive index to calculate the physical thickness. In the curvature extraction process, the image gray stretching method (IAH-GS) and the preprocessing operation are adjusted to solve the strong reflection problem of the film layer. The window threshold cutoff algorithm therein realizes defects and boundaries in OCT images with good stability. The experiments assessed the viability of employing a broadband infrared light source and a scanning objective for detecting standard elements. Finally, the thickness, curvature, and defect information of multilayer optical thin film structured lenses validate the method.

## 2. Materials and Methods

### 2.1. Materials

**Figure 1:**
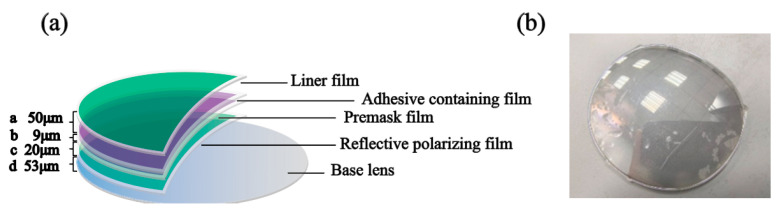
Optical film structure and physical image: (**a**) structure of lens with multilayer optical film; (**b**) physical photograph of lens placed on experimental platform.

In this study, custom-made lenses with four optical films were used for the experiments. The refractive index of the lens material group was 1.5732 at a center wavelength of 840 nm, with the specific thickness shown in Figure 1a. The surface of the base lens consists of four layers of optical films, where the a layer is the substrate film, the b layer is the adhesive-containing film, the c layer is the premask film, and the d layer is the reflective polarizing film; the actual picture is shown in Figure 1b. The experiment involved OCT scanning of the lens using an appropriate power level to ensure an optimal signal-to-noise ratio. 

### 2.2. System Structure

**Figure 2:**
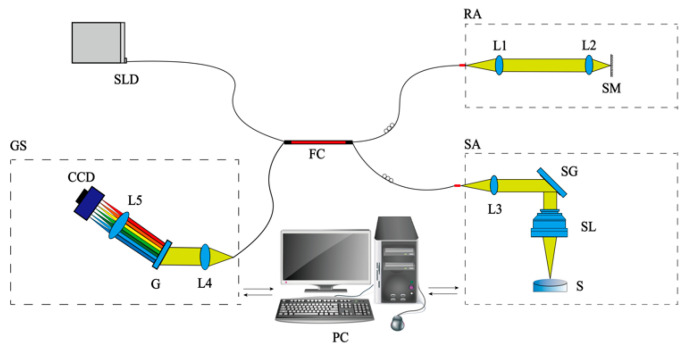
A schematic diagram of the fiber optic SD-OCT system. SLD, superluminescent diode; FC, fiber coupler; L, lens; SL, scan lens; SG, scanning galvanometer; SM, silver mirror; G, grating; CCD, charge-coupled device; S, sample; RA, reference arm; SA, sample arm; GS, grating spectrometer.

We employed the SD-OCT system to examine optical films. Figure 2 illustrates the schematic diagram of the fiber optic SD-OCT system. This system adopts a Michelson interferometer architecture and comprises a superluminescent diode (SLD), fiber coupler (FC), sample arm (SA), reference arm (RA), grating spectrometer (GS), and personal computer (PC). The broadband spectrum emitted by a super luminescent diode light source (SLD) (BLM2-D-840-B-10, Superlum, Cork, Ireland) with a central wavelength of 840 nm and a bandwidth of 90 nm was divided into the sample and reference light using a fiber coupler. The reference light was collimated into parallel light through lens L1 (AC050-008-B-ML, Thorlabs, Newton, NJ, USA). It was then focused through lens L2 (AC254-050-B-ML, Thorlabs) on the upper surface of a reflector. Finally, the reference light was reflected back into the FC. Similarly, the collimated sample light was deflected through a two-dimensional scanning galvanometer SG (GVS002, Thorlabs), focused onto an optical film by a scanning objective SL (LSM54-850, Thorlabs), and carried information about the optical paths on the upper and lower surfaces of the optical film in different regions back to the FC (TW850R5A2 50:50, Thorlabs). The difference between the optical length from FC to mirror and the optical length from FC to S is within the coherence length of the source, satisfying the interference conditions. The sample light and the reference light modulated by the film produce an interference signal in the fiber channel. Finally, the interference signals were acquired by a GS (CS800-840/80-80-OC2k-CL, Wasatch Photonics, Logan, UT, USA) and processed by a PC.

The bandwidth of the GS is 80 nm, so the corresponding theoretical axial resolution based on the wavelength of the light source is 3.88 µm. The 2D camera in GS has a pixel size of 2048 × 2048 and an A-scan speed of 70 kHz. The theoretical imaging depth of the OCT system is calculated as follows:

(1) $$Z_{max}=\frac{\lambda_{0}^{2}}{4n\delta \lambda }$$

where $Z_{max}$ represents the maximum imaging depth, $\lambda_{0}$ represents the center wavelength of the light source, n represents the refractive index of the sample, and δλ represents the resolution of the spectrometer. With a center wavelength of 840 nm and an air refractive index of 1, the calculated theoretical maximum imaging depth is 4.515 mm. We placed the mirror on the sample arm and moved the displacement controller in the reference arm to fine-tune the optical range difference. A difference of 572 pixels corresponds to a 1.259 mm shift of the reference arm. Based on the overall number of pixels, the actual maximum imaging depth can be calculated to be 4.508 mm, which is consistent with the theoretical value. In order to ensure that the system achieves the best measurement results, the optical energy of each part of the system is configured and calculated. The sensitivity and signal-to-noise ratio are often used to evaluate the imaging performance of OCT [32]. The sensitivity is defined as the maximum measurable attenuation of the detected beam which is calculated as follows:

(2) $$S[dB]=10\times \log(\frac{\sum N_{s}\times N_{ref}}{N_{el}^{2}+N_{rin}^{2}{+N}_{sh}^{2}})$$

Here, $\sum N_{s}$ denotes the sum of electrons over the entire array generated by sample arm light returning from a 100% reflector, $N_{ref}$ is the number of electrons per pixel generated by the reference arm light, and $N_{el,rin,sh}$ denote the number of electrons representing electrical noise, relative intensity noise, and scattered particle noise, respectively. We can calculate the optimal signal-to-noise ratio when $N_{rin}=N_{el}$ [33]. Calculated from the camera manual used in this system at a detection power of 0.001 W, the theoretical optimum signal-to-noise ratio of 96.85 dB was obtained at a 5.98 × 10^−6^ W reference arm energy. Based on the measurement procedure in the literature, we calculate the actual signal-to-noise ratio to be 96.08 dB.

### 2.3. Extraction Method of Thickness, Curvature, and Defects

**Figure 3:**
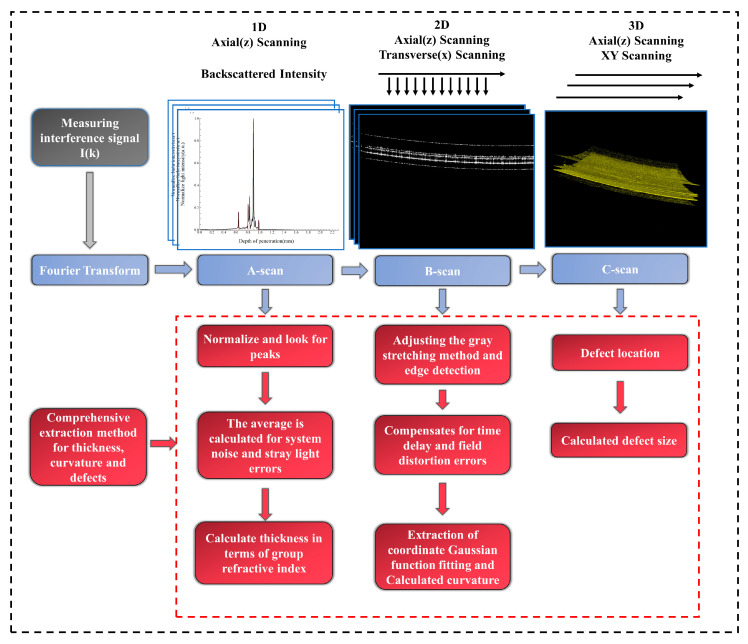
Flow of comprehensive OCT-based extraction method for thickness, curvature, and defects.

In this section, we will outline the use of SD-OCT to extract film thickness, curvature, and defects in optical components. The flowchart illustrating this method is depicted in Figure 3. 

**Figure 4:**
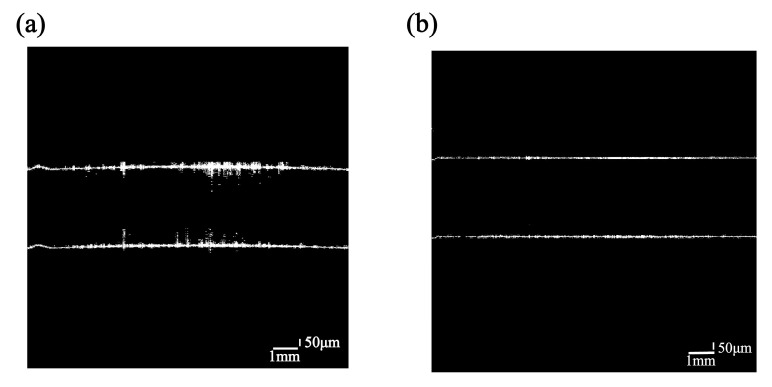
Comparison of A-scan latency and aberration before and after correction of flat glass OCT images: (**a**) glass plate image before correction; (**b**) glass plate image after correction.

The SD-OCT system conducts cross-sectional and volumetric imaging through the assessment of backscattered light intensity and time delay. Initially, the method involves isolating signal peaks from the axially scanned OCT A-scan signals within the spatial domain. The difference between the signal peaks represents the optical thickness between the film layers. In order to obtain the physical film thickness, we choose the group refractive index to calculate the actual physical thickness of the film. This also solves the problem of color dispersion associated with the use of broadband light sources in OCT. The film thickness is determined by averaging multiple measurements taken at the same position, which serves to mitigate errors arising from random noise and stray light within the experimental setup. Continuous axial measurements of 1D images, also referred to as A-scans, are conducted to determine time delays, facilitating the creation of 2D cross-sectional images known as B-scans. The B-scan can be displayed in grayscale to visualize the internal structure of the sample. However, the scanning objective is susceptible to errors across the entire field of view due to A-scan time delays and field curvature distortion, impacting our capacity to accurately fit the calculated curvature. To address this issue, we corrected for field curvature and aberrations by utilizing OpticStudio (OpticStudio 2019; Zemax, LLC, Kirkland, WA, USA) to model the error correction process. Firstly, we simulated the passage of light through the scanning objective and recorded the position of the light coordinates under the influence of field curvature and distortion aberration. Secondly, we acquire images of a standard glass plate with a specified number of scanning points and record its coordinate positions. Third, the OCT information of the samples was collected. The corresponding B-scan images were recalibrated to a standard glass plate. Finally, the coordinate bias generated by the simulation in the software (OpticStudio 2019; Zemax, LLC, Kirkland, WA, USA)is compensated into the coordinates of the image [34]. Specific examples are shown in Figure 4.

In practice, we also take into account the strong reflections from curved surfaces. During data processing, we use the individual A-scan averages for histogram-equalized grayscale stretching (IAH-GS) to minimize the effect of reflections on the surface vertices. The main process of IAH-GS is that after the acquired spectral information is Fourier-transformed, the obtained image values need to be stretched to the corresponding grayscale. At this time, the mean value of each A-scan itself is used, and finally, linear grayscale stretching is carried out. Subsequently, we extracted the coordinate values from the OCT B-scan image. The radius of curvature values was determined by fitting a Sum of Gaussians (SoG) function. Three-dimensional images (C-scan) were generated by capturing consecutive 2D images. Since C-scan data contain comprehensive information about the structure of the sample, the location of localized defects is searched for on the OCT C-scan image and the dimensional size of the defect is calculated. The software used for data acquisition processing and display of the SD-OCT system was developed in C# 6.0. Image processing and model fitting were run in MATLAB (MATLAB 2018b, The MathWorks, Inc., Natick, MA, USA).

#### 2.3.1. Principle of Thickness Extraction

**Figure 5:**
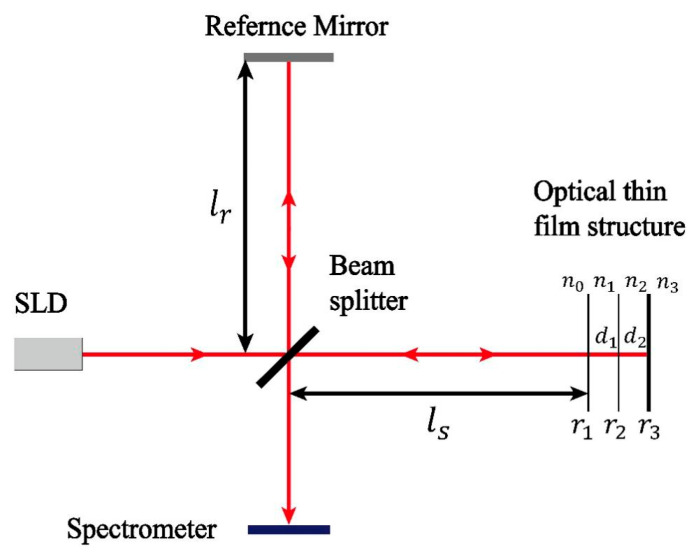
Schematic of spectral-domain OCT in optical thin film imaging.

It is assumed that two layers of optical film are present in the sample, as shown in Figure 5. 

The thickness was examined with SD-OCT. The electric field emitted by a broadband light source can be viewed as a superposition of plane waves. The electric field of a plane wave with wave number k is written as $E_{s}(k,t)$. It is divided into two parts by the beam splitter and propagates to the reference arm and the sample arm, respectively. The response $E_{r}(k)$ associated with the electric field $E_{s}\left(k,t\right)$ following its propagation through the reference arm can be written as follows:

(3) $$E_{r}(k)=\frac{1}{2}r\cdot exp(i2n_{0}kl_{r})$$

where r is the reflectance of the mirror, $n_{0}$ is the refractive index of air, k is the number of waves, and $l_{r}$ is the length of the reference arm. The response $E_{s}(k)$ of its sample arm can be derived as follows:

(4) $$E_{s}(k)=\frac{1}{2}(r_{1}+r_{2}(1-r_{1}^{2})\cdot \exp(i2n_{1}kd_{1})+r_{3}(1-r_{1}^{2})(1-r_{2}^{2})\cdot \exp(i2n_{1}kd_{1}\;+i2n_{2}kd_{2}))\cdot exp(i2n_{0}kl_{s})$$

where $n_{1}$ and $n_{2}$ represent the refractive indices of membrane layer I and membrane layer II, respectively; $d_{1}$ and $d_{2}$ represent the thickness of membrane layer I and membrane layer II; and $l_{s}$ is the length of the sample arm; $r_{1}$ and $r_{2}$ represent the reflectance at the interfaces of air and membrane layer I and membrane layer I and membrane layer II, respectively. In the spectral domain, the interference spectrum between the sample light and the reference light can be expressed as follows:

(5) $$I(k)={\left|E_{r}(k)\right|}^{2}+{\left|E_{s}(k)\right|}^{2}+2\cdot E_{r}(k)\cdot E_{s}(k)\cdot cos(2\cdot k\cdot \left|l_{s}-l_{r}\right|)$$

$\left|l_{s}-l_{r}\right|$ is the difference in path length between the signal arm and the reference arm of the interferometer. The first term in Equation (3) is the DC term, which represents the intensity of the plane mirror reflection in the reference arm. The second term is an autocorrelation term, which represents the superposition of signals reflected from different layers in the sample. Usually, this signal frequency is low (indicating a small optical path difference) and weak in intensity (as the reflectivity is much lower than that of a plane mirror, and even smaller after squaring). The third term represents the interrelated signals, which also contain valid information. The reflectivity of each layer is modulated by the optical field of the reference arm to form an interference signal. Performing an FFT on the interference spectrum converts it into the spatial domain. However, the OCT A-scan provides the light-travel information nd. Thickness calculations are relative, so time delays and aberrations do not affect the calculation of thickness from A-scan information. 

#### 2.3.2. Principle of Curvature Extraction

Curvature information is extracted from B-scan images, where optical films show significant horizontal deviation in two-dimensional cross-sectional scans. This curvature information can be calculated similarly to how OCT is used in ophthalmology [35]. Strong scattering, inherent defects in the optics, coherent scattering noise, and time delays in OCT imaging can cause artifacts. The quality of raw OCT images is often poor. This makes it difficult to accurately describe information related to the thickness, curvature, and defects of the sample. Strong reflections from smooth element surfaces may lead to saturation artifacts and loss of useful layer information. In order to avoid this as much as possible, we use the average of the individual A-scan grayscale stretching (IAH-GS) and subsequent image processing during data processing. This avoids to some extent the influence of reflections on SD-OCT images.

**Figure 6:**
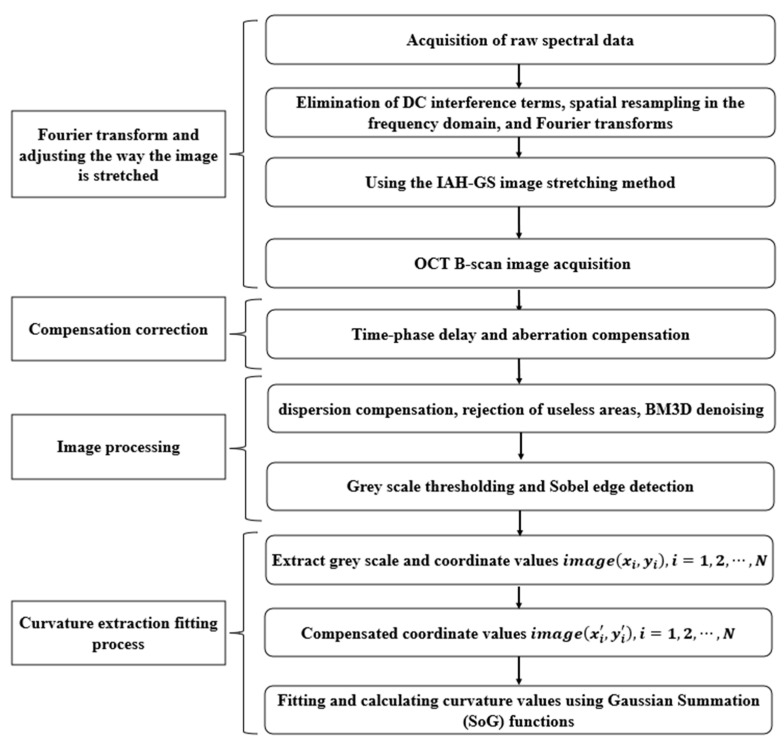
Flowchart of curvature fitting calculation algorithm.

The main steps from raw spectral data to curvature fitting calculations are shown in Figure 6. It is divided into four main parts: firstly, the Fourier transform and adjustment of the image stretching method; secondly, the compensation correction; then, the image processing; and finally, the curvature extraction fitting process.

The raw spectra are firstly subjected to operations such as removal of DC interference terms, spatial resampling in the frequency domain, and Fourier transform, and images with reduced reflection scattering effects are obtained using the IAH-GS algorithm. Next, time delay and aberration compensation corrections are performed. Then, image processing operations are performed which include dispersion compensation, elimination of image regions containing useless information, BM3D noise reduction, image grayscale stretching, and Sobel edge extraction contouring. The image quality is enhanced without losing any of the image information. Finally, curvature fitting is computed using the SoG method for the image coordinates in the image.

The coordinate information of the final 2D cross-sectional OCT B-scan image is denoted as $image(x_{i},y_{i})$, where i=1,2,⋯,N. $x_{i}$ and $y_{i}$ represent the coordinate values of the image. N represents the number of horizontal scanning points. $image(x_{i},y_{i})$ represents the intensity gray value of the image. For a given coordinate data $\left(x_{i},y_{i}\right)$, the Gaussian function model is satisfied:

(6) $$\hat{y}(x_{i})=y_{max}\;*\;exp[-\frac{{\left(x_{i}-x_{max}\right)}^{2}}{S}]$$

where $y_{max}$, $x_{max}$, and S represent the peak height, peak position, and half-width value of the Gaussian curve, respectively. The parameters in the Gaussian function are calculated by substituting the coordinates $\left(x_{i},y_{i}\right)$ into the Gaussian fitting formula [36,37]. According to the objective of the principle of least squares fitting, the three parameters of the Gaussian function are derived such that the following expression is minimized in value:

(7) $$J=min\sum_{i=1}^{N}{\left[y_{i}-\hat{y}(x_{i})\right]}^{2}$$

The final expression for the parameters of the best-fit curve is an SoG (Sum of Gaussians) function with three summation terms; each summation term consists of three fitting parameters: $a_{i}$, $b_{i}$, and $c_{i}$.

(8) $$f\left(x\right)=\sum_{i=1}^{3}a_{i}\cdot e^{-{\frac{\left(x-b_{i}\right)}{c_{i}}}^{2}}$$

The radius of curvature at any point can be determined using the curvature formula.

(9) $$K=\frac{1}{R}=\frac{\frac{d^{2}f(x)}{dx^{2}}}{{\left(1+({\frac{df(x)}{dx})}^{2}\right)}^{\frac{3}{2}}}$$

*f*(*x*) is the expression of the fitted Gaussian function, *K* represents the curvature, and *R* is the value of the radius of curvature corresponding to the curvature.

#### 2.3.3. Principle of Defect Extraction

**Figure 7:**
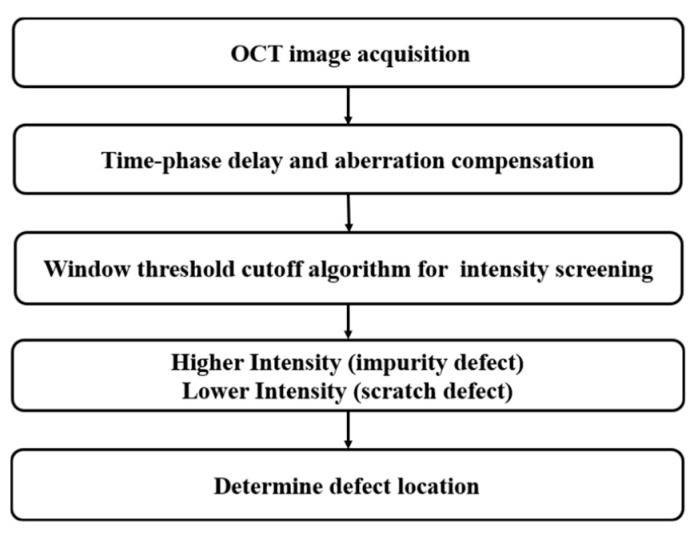
Flowchart of algorithm for automatic defect detection.

The location of the defect in the sample corresponds to a different optical path length. These optical path differences appear in the interference spectrum as cosine components with different frequencies [38]. A-scan average intensity distribution plots for different layers can reveal defective regions in the corresponding layers. All cross-sectional images are gray-stretched to identify defect locations. These defects are then displayed in the 3D OCT C-scan image of the sample. Figure 7 provides a better understanding of the defect detection principle algorithm. Firstly, standard OCT images were acquired and subjected to denoising and time delay compensation. Half of the average intensity value of the image was used as the intensity level for classification. Intensities above the image threshold can be characterized as defective areas (impurities), whereas locations with lower image intensity indicate scratch defects in the image. As a first step in the detection procedure, the tracking method is expanded horizontally and vertically [39].The algorithm is capable of rigorously determining the exact location of the defective area. After a complete scan of the sample, cross-section and surface information can be traced to the exact location of the defect.

## 3. Results and Discussion

### 3.1. System Performance Parameter Measurement

**Figure 8:**
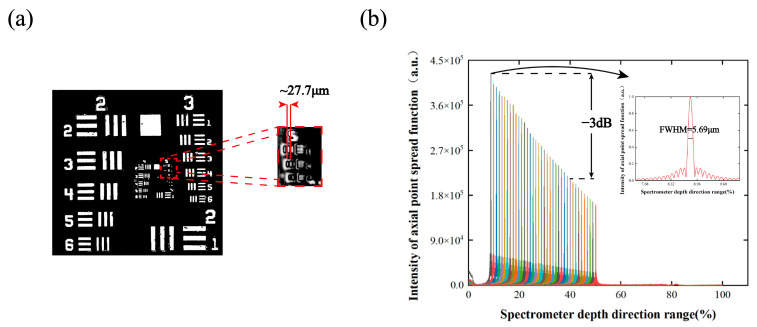
SD-OCT system performance test graph: (**a**) lateral spatial resolution characterization using USAF 1951 resolution test target; (**b**) sensitivity roll-off and axial resolution plots.

The horizontal resolution was tested using scanning standard USAF 1951 test objectives (1951 USAF Resolution Test Targets, Thorlabs, Newton, NJ, USA), as shown in Figure 8a. The system is capable of resolving elements 1 and 2 of group 5 with a lateral resolution of approximately 27.7 µm, which is generally consistent with the theoretical values.

We verified the performance of the system by placing reflective mirrors with silver-plated protective surfaces as samples in the system. The main focus was to determine the actual axial resolution, sensitivity, and sensitivity roll-off properties of the system. The system signal-to-noise ratio remains good as the imaging depth of the system increases. The recovery of real sample data is better, facilitating the system’s penetrating imaging of the lens structure. The measured axial interference intensity decay curves are shown in Figure 8b. The axial resolution in intensity (FWHM) was assessed from the half-height width of the point spread function (PSF) in the roll-off map. It is 5.69 µm in air, which is 32% larger than the theoretical value.

### 3.2. Measurement of Standard Components

In order to validate the accuracy of the systematic approach, three standard planar glass panes of varying thicknesses and a commercially available achromatic doublet lens (AC254-030-B-ML, Thorlabs) were selected. The data information of each standard part is shown in Table 1. Optically flat glass d1 and d2 are made of EAGLE XG, while d3 is made of JGS quartz. Their group refractive indices at a wavelength of 840 nm were calculated from the Cauchy dispersion equation. The effects on the thickness test are within the allowable error of the test result data. The SD-OCT system was used to perform 1024 axial OCT scans of the same area of the standard sample. The scanning field of view was adjusted to match the size of the sample, ensuring that the sampling interval was less than half the resolution.

**Figure 9:**
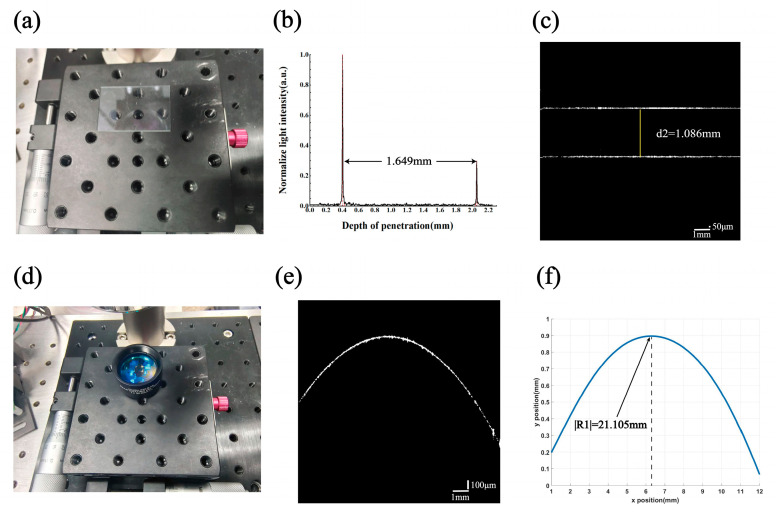
Measurements on optical flat glass d2 and double-glued lens surface R1: (**a**) optically flat glass d2 placed on the experimental platform; (**b**) A-scan information of optically flat glass d2; (**c**) B-scan image of the corrected and compensated optical plate glass d2, labeled with the actual thickness information divided by the group refractive index; (**d**) double-glued lens surface R1 placed on the experimental platform; (**e**) B-scan image of the corrected and compensated double-glued lens surface R1; (**f**) value of radius of curvature at the highest point of the R1 curve information for SoG-fitted bimodal lens surfaces.

The experimental results are shown in Figure 9. The presence of two distinct scattering peaks in the one-dimensional OCT A-scan in Figure 9b indicates the upper and lower surface locations of the glass plate d2. The difference in the transverse coordinates of these two scattering peaks is calculated and then divided by the group refractive index of the corresponding sample to determine the actual thickness value of the film d2, as shown in Figure 9b,c. For the computational measurement of the radius of curvature R1, the compensated correction method described in this paper was used. The SoG function was fitted to the points on the OCT B-scan of the 2D cross-section in Figure 9e, and the radius of curvature value was calculated as shown in Figure 9f.

The measured mean and actual values for all standard thickness samples are presented in Table 2. The measurement accuracy was calculated by substituting 1024 scans into the RMSE formula, and the final measurement accuracy was less than 1.924 µm. We have mitigated the system’s inherent errors, such as random noise and stray light, by averaging the measurements. Other errors are attributed to variations in the group refractive index caused by material impurities and inhomogeneities. We used vernier calipers to average the actual thickness measurements. However, limited by the accuracy of the reference vernier caliper, the accuracy of the actual calculated error could be further improved.

**Figure 10:**
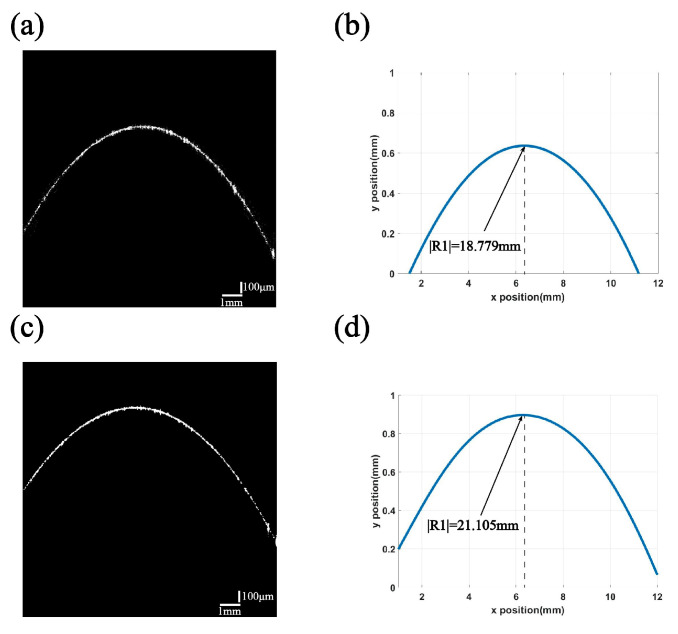
R1’s raw OCT image and R1’s corrected OCT image with calculated fitted curvature: (**a**) raw OCT image of the double-glued lens surface R1; (**b**) calculated SoG-fitted curvature of the original OCT image of the bi-adhesive lens surface R1; (**c**) R1 OCT image of a double-glued lens surface corrected for A-scan time delay and aberration; (**d**) calculated SoG-fitted curvature of the corrected OCT image of the bilayer-glued lens surface R1.

Figure 10 shows the measurement results before and after the correction of R1 on the surface of a double-glued lens.

The measured mean and nominal values for all standard curvature samples are presented in Table 3. After the A-scan and aberration compensation for image coordinates, the errors of the results tested in this experiment are within 1% of the nominal values.

As the curvature of the double-glued lens optical film increases, the measurement error also increases. We have compensated for the effects of time delays from the A-scan, as well as field curvature and aberrations, using scanning objectives and correction algorithms. Additionally, the effect of interference dispersion on the fitted data points is eliminated through dispersion compensation. The remaining potential sources of error include those from the fitting of Gaussian functions and the manufacturer’s manufacturing errors in the samples. Overall, the measurements are accurate and feasible.

### 3.3. Measurement of Lenses with Multilayer Film Structures

**Figure 11:**
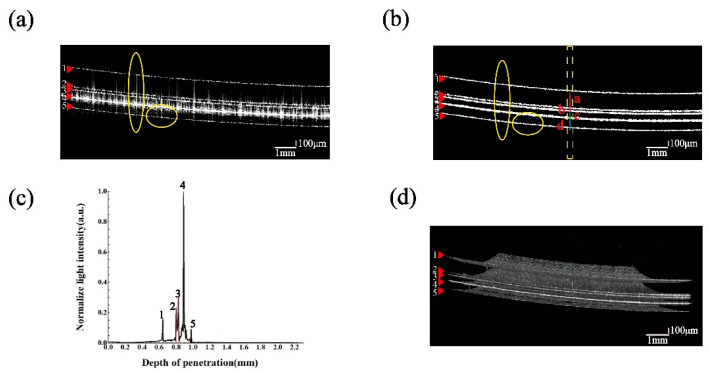
OCT information of multilayer optical thin film structured lenses: (**a**) OCT B-scan image of multilayer optical film without adjusting image stretching method and compensation (comparison of removed spikes and artifacts in yellow circles); (**b**) OCT B-scan image of multilayer optical film with adjusted image stretching and compensation (comparison of removed spikes and artifacts in yellow circles); (**c**) axial scanning information from the A-scan of the 621st pixel in the transverse direction, numbered 1 to 5, corresponds to the four-layer optical film structure; (**d**) C-scan image information of a three-dimensional optical film.

We have analyzed lenses with multilayer optical thin film structures through testing. Using a sampling interval of 10.5 µm, we reconstructed the three-dimensional morphology of film thickness and surface shape. The resulting superposition yields a cross-sectional image scan size of 2.25 mm × 12.6 mm, corresponding to a resolution of 1024 × 1200 pixels. The scanning process takes approximately 15 s to record data, and subsequent data processing to convert the data to reconstructed OCT images takes around 5 min. The results of the experiment are shown in Figure 11. As can be seen from the comparison of Figure 11a with Figure 11b, OCT image extraction of multilayer optical films does suffer from strong reflections, resulting in tampering of signals between layers. This is due to the reflective film of the fourth layer in the lens, which reflects a large number of photons and interferes with the reference arm photons, resulting in the phenomenon of CCD saturation, which destroys the imaging effect of the other layers. The morphology of the optical film itself is restored by the correction model and image processing method (IAH-GS) proposed in this paper. From the image information obtained in Figure 11c, Figure 11b, and Figure 11d (representing three OCT scans: A-scan, B-scan, and C-scan, respectively), we can determine that the surface of the lens contains four layers of optical thin film structures.

Using the A-scan information from Figure 11c, the thickness of the film layer at a corresponding scan point can be calculated. As shown in Table 4. Because of the presence of the reflective polarizing film 4 in the structure of the thin film layer, the light has to travel twice as far before reflecting the polarizing film 4, and the result we calculated from the A-scan needs to be divided twice to obtain the information about the actual thickness of the thin film layer.

**Figure 12:**
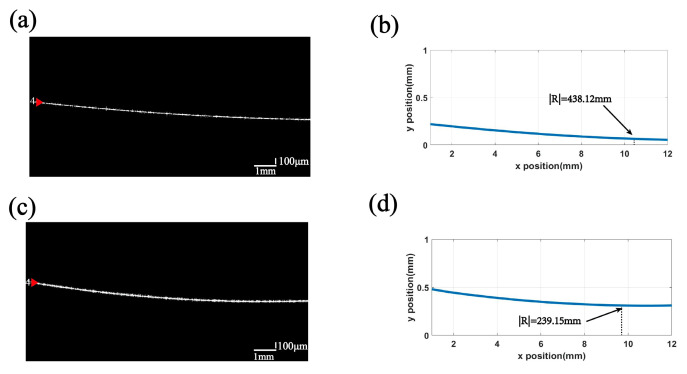
Curvature measurement of reflective film layer 4 in a multilayer optical thin film structured lens: (**a**) the raw OCT image of the reflective film layer 4; (**b**) reflected thin film layer 4 SoG-fitted calculated curvature results of the original OCT image; (**c**) OCT image of reflective film layer 4 after correction for A-scan delay and aberration; (**d**) reflected thin film layer 4 corrected OCT image of SoG-fitted calculated curvature results.

Additionally, the dispersion mismatch between the reference and sample arms, as well as the dispersive medium, leads to a widening of the coherence envelope, with the corresponding scattering peaks exhibiting a certain half-height width [40]. It can also be observed that the brightness of the optical film layer numbered 4 is the most pronounced, due to its high reflectivity. This is consistent with the actual condition of our sample. The image information of the highly reflective thin film layer in the optical thin film structure was extracted from the B-scan shown in Figure 11b. Figure 12 shows the fitting calculations before and after the correction of the OCT image. 

**Figure 13:**
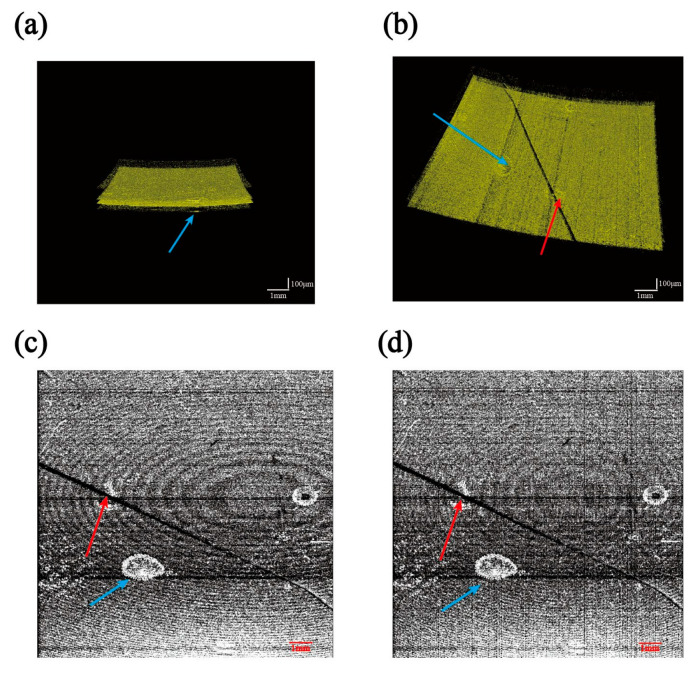
Defect location results for multilayer optical thin film structured lenses: (**a**) cross-sectional view of a defect in a C-scan image of a 3D dataset; (**b**) frontal top view of a defect in a C-scan image of a 3D dataset; (**c**) layer section view of the C-scan image of the 3D data before correction; (**d**) layer section view of the C-scan image of the 3D data after correction.

Using the fitting method described in this paper, we calculated the value of the nadir curvature. The absolute value of the radius of curvature R before correction is 438.12 mm. The average of several measurements yields an absolute value of the final corrected radius of curvature R of 239.15 mm. The nominal value of the radius of curvature of the reflective film at the measurement position is 240.00 mm. The fitting error was reduced from 82.550% to 0.354%. The main source of error is the relatively small change in the curvature of the film layer. This method can also be used to analyze the radius of curvature of other thin film layers. Figure 13 shows C-scan images of multilayer optical thin film structures with defective regions. The blue arrows in Figure 13a–d indicate bubble defects in the inner layer of the optical film, while the red arrows indicate scratch defects. 

Comparing Figure 13c,d, we can first see that there is a difference in the background interference halo due to reflected light. It was elliptical before correction. This is clearly affected by the A-scan time delay and aberrations such as field curvature distortion. The halo returned to a normal round shape after correction using our method. The information we obtain from our images at this point is much more accurate. From Figure 12d, the size of the bubble defect is calculated to be approximately 1.674 mm × 0.986 mm, and the length of the scratch defect is about 12.99 mm. The length of the scratches before calibration was 13.23 mm. The actual true value of the scratches in the scanned area is 13.00 mm. Calculation errors were reduced from 1.769% to 0.077%. Cross-sectional images obtained non-destructively with the SD-OCT system allow for the accurate identification and precise location of defects. Computer vision inspection methods can detect these defect details but cannot provide 3D information [41]. The results demonstrate that the method presented in this paper can extract chromatographic information regarding optical film thickness, curvature, and defects with high repeatability and accuracy.

## 4. Conclusions

This study demonstrates the application of SD-OCT in industry, where standard components as well as lenses with multilayer optical film structures were inspected and analyzed using the SD-OCT system. This article proposes a complete process for OCT signals as well as image processing for multilayer optical films, which solves the crosstalk problem of multilayer film signals. Ultimately, the detection of thickness and curvature is realized, and the morphology and size of film defects can also be recognized. The SD-OCT system has fast inspection speed, high precision, and a large field of view. And it can also achieve a one-time detection of thickness, curvature, and defects, which greatly improves efficiency. The curvature measurement calibration results remain within 1% of the nominal values. Therefore, SD-OCT facilitates the rapid inspection and analysis of the internal film structures of optical components, touchscreen panels, glass panels, and other transparent materials. The SD-OCT system features a simple structure and uncomplicated calculations for subsequent data processing, enabling faster, more reliable, and more comprehensive extraction of sample component information. This ensures the quality of the sample components and suggests that SD-OCT could become a new inspection method for the optical industry. Future research may expand this work to include applications in photolithography and integrated circuits. Additionally, SD-OCT monitors the alignment of layers, thickness variations, and uniformity of samples in real time, ensuring accurate performance at each step, which significantly improves productivity and product quality.

## Figures and Tables

**Table 1:** Thickness and curvature value of standardized samples.

Standard Sample	Group Refractive Index	Thickness and Curvature Value (mm)
Optically flat glass d1	1.5187	0.712
Optically flat glass d2	1.5187	1.096
Optically flat glass d3	1.4659	1.480
Double-glued lens surface R1	1.6820	21.090
Double-glued lens surface R2	1.8693	−79.080

**Table 2:** Measured mean and actual values of all standard thickness samples.

Standard Sample	Group Refractive Index	Actual Value (mm)	Measured Mean (mm)	Measurement Accuracy (µm)	Absolute Error (mm)
d1	1.5187	0.72	0.7048	1.924	−0.0152
d2	1.5187	1.10	1.0902	1.717	−0.0098
d3	1.4659	1.48	1.4793	1.582	−0.0007

**Table 3:** Measured mean and nominal values of all standard curvature samples.

Standard Sample	Nominal Value (mm)	OCT Raw Image Fitting Value (mm)	OCT Image-Corrected Fitted Value (mm)	Raw Image Fitting Error	Corrected Image Fitting Error
R1	21.090	18.779	21.105	10.958%	0.071%
R2	−79.080	−59.562	−78.292	24.681%	0.996%

**Table 4:** Measured and actual values of lenses with multilayer optical thin film structures.

Film Number	Actual Value (mm)	Measured Value (mm)	Corrected Value (mm)	Absolute Error (mm)
a	0.050	0.1018	0.0509	0.0009
b	0.009	0.0156	0.0078	−0.0012
c	0.020	0.0411	0.02055	0.00055
d	0.053	0.0548	0.0548	0.0018

## Data Availability

The data presented in this study are available on request from the corresponding author.

## References

[1] Jena S., Tokas R., Thakur S., Sahoo N. (2015). Characterization of optical thin films by spectrophotometry and atomic force microscopy. SMC Bull..

[2] Davies T.E., Li H., Bessette S., Gauvin R., Patience G.S., Dummer N.F. (2022). Experimental methods in chemical engineering: Scanning electron microscopy and X-ray ultra-microscopy—SEM and XuM. Can. J. Chem. Eng..

[3] Ren Y., Cao Z., Tang X., Xie H., Xu L. (2019). μm-resolution thickness distribution measurement of transparent glass films by using a multi-wavelength phase-shift extraction method in the large lateral shearing interferometer. Opt. Express.

[4] Nestler P., Helm C.A. (2017). Determination of refractive index and layer thickness of nm-thin films via ellipsometry. Opt. Express.

[5] Zhang R., Shi L., Zhou S., Zhang J., Liu B., Wu G. (2022). Dynamic ellipsometry measurement based on a simplified phase-stable dual-comb system. Opt. Express.

[6] Aidukas T., Phillips N.W., Diaz A., Poghosyan E., Müller E., Levi A., Aeppli G., Guizar-Sicairos M., Holler M. (2024). High-performance 4-nm-resolution X-ray tomography using burst ptychography. Nature.

[7] Jiang Y., Yuan M., Ji X., Zhang Y., Li M. (2023). Ultrasonic nondestructive evaluation of the bonding strength of polyurethane coatings based on feedforward comb filtering effect. Ultrasonics.

[8] Stock S.R. (2019). Microcomputed Tomography: Methodology and Applications.

[9] Rao C., Zhong L., Guo Y., Li M., Zhang L., Wei K. (2024). Astronomical adaptive optics: A review. PhotoniX.

[10] Drexler W., Fujimoto J.G. (2008). Optical Coherence Tomography: Technology and Applications.

[11] Wang Y., Chen S., Chen X., Xu Z., Lin K., Shi L., Mu Q., Liu L. (2024). Coaxial Bright and Dark Field Optical Coherence Tomography. IEEE Trans. Biomed. Eng..

[12] Wang Y., Chen S., Lin K., Chen X., Xu Z., Lou S., Ge X., Ni G., Yu X., Mo J. (2023). Multi-channel spectral-domain optical coherence tomography using single spectrometer. Chin. Opt. Lett..

[13] Ginner L., Kumar A., Fechtig D., Wurster L.M., Salas M., Pircher M., Leitgeb R.A. (2017). Noniterative digital aberration correction for cellular resolution retinal optical coherence tomography in vivo. Optica.

[14] Lee C., Kim K., Han S., Kim S., Lee J.H., Kim H.K., Kim C., Jung W., Kim J. (2014). Stimulated penetrating keratoplasty using real-time virtual intraoperative surgical optical coherence tomography. J. Biomed. Opt..

[15] Mujat M., Ferguson R.D., Patel A.H., Iftimia N., Lue N., Hammer D.X. (2010). High resolution multimodal clinical ophthalmic imaging system. Opt. Express.

[16] Cho N.H., Jang J.H., Jung W., Kim J. (2014). In vivo imaging of middle-ear and inner-ear microstructures of a mouse guided by SD-OCT combined with a surgical microscope. Opt. Express.

[17] Yu X., Tang H., Hu C., Ding Q., Wang L., Wang X., Fan Z., Liu L. (2018). Multiscale skin imaging in vivo using optical coherence tomography. Laser Phys. Lett..

[18] Min E., Lee J., Vavilin A., Jung S., Shin S., Kim J., Jung W. (2015). Wide-field optical coherence microscopy of the mouse brain slice. Opt. Lett..

[19] Liu P., Groves R.M., Benedictus R. (2014). 3D monitoring of delamination growth in a wind turbine blade composite using optical coherence tomography. NDT E Int..

[20] Alarousu E., AlSaggaf A., Jabbour G.E. (2013). Online monitoring of printed electronics by Spectral-Domain Optical Coherence Tomography. Sci. Rep..

[21] Wijesinghe R.E., Park K., Jung Y., Kim P., Jeon M., Kim J. (2017). Industrial resin inspection for display production using automated fluid-inspection based on multimodal optical detection techniques. Opt. Lasers Eng..

[22] Jeon D., Jung U., Park K., Kim P., Han S., Jeong H., Wijesinghe R.E., Ravichandran N.K., Lee J., Han Y. (2020). Vision-inspection-synchronized dual optical coherence tomography for high-resolution real-time multidimensional defect tracking in optical thin film industry. IEEE Access.

[23] Karimi Y., Yang H., Liu J., Park B.H., Chamanzar M. (2022). Enhanced spectral-domain optical coherence tomography (SD-OCT) using in situ ultrasonic virtual tunable optical waveguides. Opt. Express.

[24] Ozaki N., Ishida K., Nishi T., Ohsato H., Watanabe E., Ikeda N., Sugimoto Y. (2020). OCT with a Visible Broadband Light Source Applied to High-Resolution Nondestructive Inspection for Semiconductor Optical Devices. Optical Coherence Tomography and Its Non-Medical Applications.

[25] Zhang Z., Yang X., Zhao Z., Zeng F., Ye S., Baldock S.J., Lin H., Hardy J.G., Zheng Y., Shen Y. (2023). Rapid imaging and product screening with low-cost line-field Fourier domain optical coherence tomography. Sci. Rep..

[26] Dunkers J.P., Parnas R.S., Zimba C.G., Peterson R.C., Flynn K.M., Fujimoto J.G., Bouma B.E. (1999). Optical coherence tomography of glass reinforced polymer composites. Compos. Part A Appl. Sci. Manuf..

[27] Frank S., Seiler M., Bliedtner J. (2021). Three-dimensional evaluation of subsurface damage in optical glasses with ground and polished surfaces using FF-OCT. Appl. Opt..

[28] Wolfgang M., Koutsamanis I., Spoerk M. (2023). Optical Coherence Tomography as a novel tool for non-invasive membrane thickness monitoring of co-extruded drug-delivery systems. Chem. Eng. Res. Des..

[29] Ortiz S., Siedlecki D., Remon L., Marcos S. (2009). Optical coherence tomography for quantitative surface topography. Appl. Opt..

[30] Manallah A., Bouafia M., Meguellati S. (2015). Optical coherence tomography as film thickness measurement technique. Proceedings of the Photonics Devices, and Systems VI.

[31] Shirazi M.F., Wijesinghe R.E., Ravichandran N.K., Kim P., Jeon M., Kim J. (2018). Quality assessment of the optical thin films using line field spectral domain optical coherence tomography. Opt. Lasers Eng..

[32] Leitgeb R., Hitzenberger C., Fercher A. (2003). Performance of fourier domain vs. time domain optical coherence tomography. Opt. Express.

[33] Yun S., Tearney G., Bouma B., Park B., de Boer J.F. (2003). High-speed spectral-domain optical coherence tomography at 1.3 µm wavelength. Opt. Express.

[34] Tianhui R., Hongjun W., Liwei W. (2021). Development of a whole eye tissue parameter measurement system based on swept-source OCT. Med. Health Equip..

[35] Wagner J. (2020). Quantitative Measurement of the Cornea by OCT. Ph.D. Thesis.

[36] Breher K., Agarwala R., Leube A., Wahl S. (2019). Direct modeling of foveal pit morphology from distortion-corrected OCT images. Biomed. Opt. Express.

[37] Zeephongsekul P., Jayasinghe C.L., Fiondella L., Nagaraju V. (2016). Maximum-likelihood estimation of parameters of NHPP software reliability models using expectation conditional maximization algorithm. IEEE Trans. Reliab..

[38] Fercher A.F., Hitzenberger C.K., Kamp G., El-Zaiat S.Y. (1995). Measurement of intraocular distances by backscattering spectral interferometry. Opt. Commun..

[39] He J., Chen S., Yang F., Huang W., Ni G., Yang J., Huang Y., Ge X., Liu L. (2024). Non-invasive quantitative blood cell counting using optical coherence tomography. Opt. Laser Technol..

[40] Chen Y., Zhao H., Wang Z. (2009). Investigation on spectral-domain optical coherence tomography using a tungsten halogen lamp as light source. Opt. Rev..

[41] Katafuchi N., Sano M., Ohara S., Okudaira M. (2000). A method for inspecting industrial parts surfaces based on an optics model. Mach. Vis. Appl..

